# Regulation of Nrf2 by X Box-Binding Protein 1 in Retinal Pigment Epithelium

**DOI:** 10.3389/fgene.2018.00658

**Published:** 2018-12-20

**Authors:** Chen Chen, Yimin Zhong, Joshua J. Wang, Qiang Yu, Kendra Plafker, Scott Plafker, Sarah X. Zhang

**Affiliations:** ^1^Department of Ophthalmology, The Second People’s Hospital of Yunnan Province, Kunming, China; ^2^Key Laboratory of Yunnan Province for the Prevention and Treatment of Ophthalmic Diseases, Yunnan Eye Institute, Kunming, China; ^3^Department of Medicine, The University of Oklahoma, Oklahoma City, OK, United States; ^4^State Key Laboratory of Ophthalmology, Zhongshan Ophthalmic Center, Sun Yat-sen University, Guangzhou, China; ^5^Department of Ophthalmology, University at Buffalo, The State University of New York, Buffalo, NY, United States; ^6^Aging and Metabolism Research Program, Oklahoma Medical Research Foundation, Oklahoma City, OK, United States

**Keywords:** retinal pigment epithelium, NF-E2-related factor 2, X-box binding protein 1, endoplasmic reticulum stress, oxidative stress, cell death

## Abstract

Normal function of the retinal pigment epithelium (RPE) is essential for maintaining the structural integrity of retinal photoreceptors and the visual process. Sustained oxidative damage of the RPE due to aging and other risk factors contributes to the development of age-related macular degeneration (AMD). The transcription factor NF-E2-related factor 2 (Nrf2) is a central regulator of cellular antioxidant and detoxification responses. Enhancing Nrf2 function protects RPE cells from oxidation-related apoptosis and cell death. Previously, we demonstrated that Nrf2 activation can be induced by endoplasmic reticulum (ER) stress; however, the mechanisms are not fully understood. In the present study, we examined the role of X box-binding protein 1 (XBP1), an ER stress-inducible transcription factor, in regulation of Nrf2 in the RPE. We found that RPE-specific *XBP1* conditional knockout (cKO) mice exhibit a significant reduction in Nrf2 mRNA and protein levels, along with decreased expression of major Nrf2 target genes, in the RPE/choroid complex. Using primary RPE cells isolated from *XBP1* cKO mice and human ARPE-19 cell line, we confirmed that loss of *XBP1* gene or pharmacological inhibition of *XBP1* splicing drastically reduces Nrf2 levels in the RPE. Conversely, overexpression of spliced *XBP1* results in a modest but significant increase in cytosolic and nuclear Nrf2 protein levels without affecting the transcription of Nrf2 gene. Moreover, induction of ER stress by tunicamycin and thapsigargin markedly increases Nrf2 expression, which is abolished in cells pretreated with *XBP1* splicing inhibitors 4μ8C and quinotrierixin. Mechanistic studies indicate that quinotrierixin reduces Nrf2 expression likely through inhibition of protein translation. Finally, we demonstrate that overexpression of Nrf2 protected RPE cells against oxidative injury but appeared to be insufficient to rescue from XBP1 deficiency-induced cell death. Taken together, our results indicate that XBP1 modulates Nrf2 activity in RPE cells and that XBP1 deficiency contributes to oxidative injury of the RPE.

## Introduction

The retinal pigment epithelium (RPE), a monolayer of pigmented epithelial cells in the eye, plays a crucial role in the nourishment and detoxification of photoreceptors (Strauss, 2005). Dysfunction of the RPE is associated with a variety of posterior segment ocular diseases including age-related macular degeneration (AMD), the leading cause of blindness in the elderly (Bressler, 2004; Augood et al., 2006). Due to its high consumption of oxygen and continuous exposure to light, the RPE is constantly subjected to oxidative stress and its unique phagocytic function further imposes an additional oxidative burden (Cai et al., 2000). Cumulative oxidative stress also increases complement activation (Thurman et al., 2009) and upregulates pathogenic genes including VEGFA and Annexin 2 promoting drusen formation and choroidal neovascularization (Rabin et al., 2013). Anti-oxidants such as melatonin and N-Acetylcysteine (NAC) successfully alleviate RPE apoptosis (Seko et al., 2001; Fu et al., 2012) and dietary supplement of anti-oxidants including vitamin A, C, and E, β-carotene and lutein modestly reduce the progression of AMD (Tan et al., 2008; Ho et al., 2011), suggesting a critical role of oxidative stress in RPE injury and AMD.

To cope with the oxidative environment, the RPE has evolved effective defenses against oxidative stress. It is rich in antioxidants such as vitamin E, as well as enzymes and proteins that neutralize free radicals, including superoxide dismutase (SOD), catalase, and glutathione (Shamsi and Boulton, 2001). In addition, RPE cells have robust anti-oxidant and anti-stress response systems to maintain redox and proteome homeostasis in the face of high oxygen tension and photo-oxidative stress, and this system is primarily regulated by NF-E2-related factor 2 (Nrf2), a bZIP transcription factor (Kaspar et al., 2009; Niture et al., 2010). By binding to antioxidant response elements (AREs) and electrophile-responsive element (EpRE), Nrf2 activates a group of ARE-containing antioxidant genes including GSTA1, GSTA2, NQO-1, and HO-1 (Kang et al., 2005), and a host of phase II detoxification enzymes (Na and Surh, 2008). In the RPE, Nrf2 signaling regulates glutathione synthesis and protects against photooxidative damage (Gao and Talalay, 2004). Mice lacking Nrf2 develop drusen-like deposits, accumulation of lipofuscin, spontaneous choroidal neovascularization (CNV) and sub-RPE deposition of inflammatory proteins, which resemble human AMD (Zhao et al., 2011). These findings, along with compelling evidence from other published studies, support the importance of Nrf2 signaling in the anti-oxidant defense system of the RPE.

It is well established that the stability and activity of Nrf2 are directly coupled to cellular redox and proteome status (Itoh et al., 1999; Nguyen et al., 2003, 2004, 2005; Hohn and Grune, 2014). During homeostasis, the majority of Nrf2 is degraded, and this turnover is mediated by the ubiquitin proteolytic system. Specifically, Nrf2 is degraded by the multi-subunit E3 ligase, CUL3KEAP1, in cooperation with the 26S proteasome (Kobayashi et al., 2004, 2006; Li and Kong, 2009). Oxidative or proteotoxic stresses, however, dissociate CUL3KEAP1, resulting in the stabilization of Nrf2 and its translocation into the nucleus to induce the transcription of anti-oxidant and chaperone genes, estimates of which number in the hundreds (Kang et al., 2005; Na and Surh, 2008).

XBP1 is a member of the CREB/ATF basic region-leucine zipper family of transcription factors. It’s a central regulator in unfolded protein response (UPR) and endoplasmic reticulum stress (ER stress). Under ER stress, activated ER membrane sensor IRE1 splices a 26 bp fragment from XBP1 mRNA, resulting in the conversion of a 267 amino acid unspliced XBP1 protein to a 371 amino acid spliced XBP1 protein (Glimcher, 2010). The spliced XBP1 then translocates into the nucleus and regulates the expression of its downstream genes. In previous studies, we found that induction of ER stress by cigarette smoking, a major environmental risk factor for AMD, activates the UPR and increases Nrf2 expression in the RPE (Chen et al., 2014; Huang et al., 2015a,b). Deletion of C/EBP homolog protein 10 (CHOP) suppresses Nrf2 upregulation resulting in exacerbated cell death, suggesting a role of UPR in Nrf2 regulation (Huang et al., 2015b). In addition, ablation of XBP1 in the mouse RPE results in increased reactive oxygen species (ROS) and RPE apoptosis (Zhong et al., 2012; Chen et al., 2014); conversely, overexpression of *XBP1* protects RPE cells from cigarette smoke exact or hydroquinone induced cell death (Chen et al., 2014; Huang et al., 2015b). These results suggest a role of XBP1 in regulation of oxidative stress in RPE cells. In the present study, we investigate whether XBP1 regulates Nrf2 expression in the RPE and explore the underlying mechanism.

## Materials and Methods

### Animals

Generation of RPE-specific *XBP1* conditional knockout (cKO) mice was described elsewhere (Zhong et al., 2012; Chen et al., 2014). Littermate mice (*XBP1* floxed, Cre-) were used as control in all experiments. Mice were maintained on a 12 h light/12 h dark cycle with *ad libitum* access to food and water. All animal procedures were approved by the Institutional Animal Care and Use Committees at the University of Oklahoma Health Sciences Center and the University at Buffalo, State University of New York, and in accordance with the ARVO statements for the Use of Animals in Ophthalmic and Vision Research.

### *Ex vivo* Eyecup Incubation

Eyecups containing RPE, choroid, and sclera were incubated with 10 mg/ml tunicamycin for 6 h. Proteins were extract from the RPE by incubation of lysis buffer with the inner surface of the eyecups for 30 min and then subjected to Western blot analysis.

### Immunohistochemistry of Mouse Retina

For frozen sections, the cornea and lens were removed and the eyecups were fixed with 4% paraformaldehyde for 30 min. Eyecups were then cryoprotected with a series of sucrose solution (10–30%) and cross-sections of the retina were obtained using a cryostat. Retinal sections were immunostained using anti-Nrf2 antibody (1:100; Santa Cruz Biotechnology) overnight at 4°C, followed by biotinylated secondary antibody and fluorescein isothiocyanate avidin. The fluorescence was visualized under an Olympus AX70 microscope.

### Primary Mouse RPE Cell Culture

Primary RPE cells were isolated from *XBP1*^flox/flox^ and *XBP1* RPE specific knock-out mouse pups as previously described (Gibbs et al., 2003) with modifications. Briefly, 14 days pups were sacrificed by cervical dislocation, eyeballs enucleated immediately. Eyes were washed with Dulbecco’s Modification of Eagle’s Medium (DMEM)/Ham’s F-12 50/50 mix medium (Cellgro, Manassas, VA, United States), and digested with 2% (wt/vol) dispase (GIBCO, #17105-041) in serum-free DMEM/F12 at 37°C for 45 min. Digested eyeballs were transferred to a 60 mm culture dish containing growth medium [DMEM/F12 with 10% fetal bovine serum (FBS), 1% penicillin/streptomycin] and dissected under a surgical microscope. Anterior segments and neural retinas were removed and the single sheets of RPE cells were peeled off the eyecup and collected. RPE layer was digested with 0.05% trypsin and the resulting singe cells of RPE were seeded in 12-well plate in growth medium, and allowed 7–10 days to grow until confluence before used for experiments.

### ARPE-19 Cell Culture

Human RPE (ARPE-19) cells were purchased from American Type Culture Collection (ATCC, Manassas, VA, United States) and maintained in DMEM/F12 medium containing 10% FBS and 1% antibiotic/antimycotic. Cells were allowed to grow to 100% confluence and quiescent overnight with serum free DMEM/F12 medium before adding all chemicals.

### Chemicals

Tunicamycin (TM) and thapsigargin (TG) were purchased from EMD Millipore Corporation (Billerica, MA, United States); Hydroquinone (HQ), tert-Butylhydroquinone (tBHQ), and cycloheximide (CHX) were purchased from Sigma-Aldrich (St. Louis, MO, United States); MG132 was purchased from Cayman Chemical (Ann Arbor, MI, United States). 4μ8C was provided by Dr. David Ron (University of Cambridge Metabolic Research Laboratories and National Institute for Health Research Cambridge Biomedical Research Centre, United Kingdom); Quinotrierixin (QT) was a kind gift from Dr. Etsu Tashiro (Keio University, Japan).

### Adenoviral Transduction and siRNA Transfection

Construction of adenovirus expressing Nrf2 and adenoviral transduction were described previously (Huang et al., 2015b). Briefly, ARPE-19 cells were transduced with adenoviruses overexpressing either spliced *XBP1, Nrf2*, or *LacZ* (transfection control) at a multiplicity of infection (MOI) of 10 and 20 for Ad-*XBP1s* and a MOI of 50 and 100 for Ad*-Nrf2*. To knock down *XBP1* (both unspliced and spliced forms), ARPE-19 cells were transfected with *XBP1* siRNA or control siRNA (Santa Cruz Biotechnology, Santa Cruz, CA, United States) using Lipofectamine 2000 (Invitrogen, Carlsbad, CA, United States) per manufacturer’s instruction. 24 h after transduction/transfection, cells were quiescent overnight with serum free DMEM/F12 medium followed by desired treatments.

### Western Blot Analysis

Mouse eyecups or ARPE-19 Cells were lysed in RIPA buffer with protease inhibitor cocktail, PMSF and sodium orthovanadate (Santa Cruz Biotechnology) and sonicated by ultrasound (on ice). Protein concentration was quantified using BCA kit (Pierce Biotechnology, Inc., Rockford, IL, United States). Twenty-five micrograms of protein were resolved by SDS-PAGE gel and electro-transferred to nitrocellular membranes. After blocking with 5% non-fat milk, membranes were blotted overnight at 4°C with following primary antibodies: anti-Nrf2 (1:1000; Santa Cruz Biotechnology), anti-XBP1 (1:500; Santa Cruz Biotechnology), anti-Catalase (1:2000; Sigma-Aldrich), anti-SOD1 (1:2000; Abcam), anti-SOD2 (1:1000; Assay Designs, MI, United States) and anti-β-actin (1:5,000; Abcam). After incubation with HRP-conjugated secondary antibodies, membranes were developed with chemiluminescence substrate (Thermo Fisher Scientific, Rockford, IL, United States: #34076) using Vision Works LS image acquisition and analysis software (UVP, Upland, CA, United States), and bands were semi-quantified by densitometry.

### RT-PCR and Real-Time RT-PCR

For ARPE-19 cells, total RNA were extracted using an E.Z.N.A. total RNA kit I (Omega Bio-tek, Georgia, GA, United States) and 1 μg RNA was used to synthesize cDNA. For mouse eyecups, total RNA were extracted using Trizol reagent (Invitrogen, Grand Island, NY, United States) and 200 ng RNA was used to synthesize cDNA. The Maxima First Strand cDNA synthesis kit containing oligo (dT) and random hexamer primers (Fermentas, Glen Burnie, MD, United States) was used for cDNA synthesis. To investigate *XBP1* splicing, RT-PCR was performed using the cDNA template and PCR Master Mix (Fermentas, #K1081), and PCR products were resolved on a 2.5% agarose/1 × TAE gel. The RT-PCR primers for *human XBP1* were: 5′-TTACGAGAGAAAACTCATGGC-3′, 5′-GGGTCCAAGTTGTCCAGAATGC-3′ (Lin et al., 2007). Real-time quantitative RT-PCR was performed using SYBR^®^ Green PCR Master Mix (Bio-Rad Laboratories, Hercules, CA, United States) and primers were listed in Table 1. The mRNA levels of target genes were normalized by 18s ribosomal RNA.

**Table 1:** Primers used for quantitative real-time PCR.

Gene name	Primer sequence
*Mouse XBP1 (exon2)*	5′-CCTGAGCCCGGAGGAGAA-3′
	5′-CTCGAGCAGTCTGCGCTG-3′
*Mouse Nrf2*	5′-AGGACATGGAGCAAGTTTGG-3′
	5′-TCCTCAAAACCATGAAGGAA-3′
*Mouse NQO-1*	5′-AGGGTTCGGTATTACGATCC-3′
	5′-AGTACAATCAGGGCTCTTCTCG-3′
*Mouse HO-1*	5′-TCTATCGTGCTCGCATGAAC-3′
	5′-CTGTCTGTGAGGGACTCTGG-3′
*Mouse GST*	5′-TCTGCCTATATGAAGACC-3′
	5′-AGAGAAGTTACTGGAAGC-3′
*Human total XBP1*	5′-CCATGGATTCTGGCGGTATTGACT-3′
	5′-CCACATTAGCTTGGCTCTCTGTCT-3′
*Human spliced XBP1*	5′-CCGCAGCAGGTGCAGG-3′
	5′-GAGTCAATACCGCCAGAATCCA-3′
*Human Nrf2*	5′-AAAGAGCGCCGAGGATTTCAG-3′
	5′-CCAAGAAATGCAGTCTCGAG-3′
*Human Erdj4*	5′-CTGTATGCTGATTGGTAGAGTCAA-3′
	5′-AGTAGACAAAGGCATCATTTCCAA-3′
*Human p58^IPK^*	5′-GAGGTTTGTGTTGGGATGCAG-3′
	5′-GCTCTTCAGCTGACTCAATCAG-3′
*Human HO-1*	5′-CAGTGCCACCAAGTTCAAGC-3′
	5′-GTTGAGCAGGAACGCAGTCTT-3′

### Nrf2 Half-Life Experiments

2 × 10^5^ ARPE19 cells were plated/well in 3 cm dishes. Two days after plating, cells were treated with QT (0.5 μM) or vehicle (DMSO) for 7 h. Cells were then starved of methionine and cysteine with or without 10 μM MG132 for 30 min before the addition of 35S-translabel for an additional 40 min. The radiolabeled media was removed, cells were washed with ice-cold PBS, and then warm media was added (+/- QT) for 0, 15, 30, or 60 min before endogenous Nrf2 was immunoprecipitated from the cell lysates, resolved by SDS-PAGE and the dried down gels were processed for fluorography, all as described previously (Plafker et al., 2010).

### Nrf2 Stabilization by MG132

ARPE19 cells were treated with QT or vehicle for 7 h and subsequently pulsed for 40 min with 35S-translabel in the presence or absence of MG132. Cells were then immediately lysed under denaturing conditions and processed for immunoprecipitation of endogenous Nrf2 as described for the half-life experiments.

### TUNEL Assay

ARPE19 cell DNA fragmentation was detected using the *In Situ* Cell Death Detection TMR red kit (TUNEL Assay, Roche Diagnostics Corp., Indianapolis, IN, United States) according to manufacturer’s protocol. Briefly, cells on coverslips were fixed with 4% PFA for 1 h, followed by permeabilization for 2 min on ice in 0.1% citrate buffer containing 0.1% Triton X-100. Then coverslips were incubated at 37°C in TUNEL reaction mix containing nucleotides and terminal deoxynucleotidyl transferase (TdT). Incubation without TdT enzyme was conducted as a negative control. After three washes with PBS, coverslips were mounted to a slide with a mounting medium for fluorescence with DAPI (Vector Laboratories, Burlingame, CA, United States. #H-1200), and observed under a fluorescence microscope.

### Statistical Analysis

The quantitative data were expressed as mean ± SD. Statistical analyses were performed using Student’s *t*-test for two-group comparisons and one-way ANOVA with Bonferroni’s multiple comparison tests for three groups or more. Statistical differences were considered significant at a *P*-value of less than 0.05. When quantifying Western Blots for ARPE-19 cells, the ratio of the target protein to β-actin in control group was set as 1.0 in all experiments, and the ratios of the target protein to β-actin in other groups were expressed as folds of control.

## Results

### Ablation of *XBP1* Reduces Nrf2 Expression in the RPE

**FIGURE 1:**
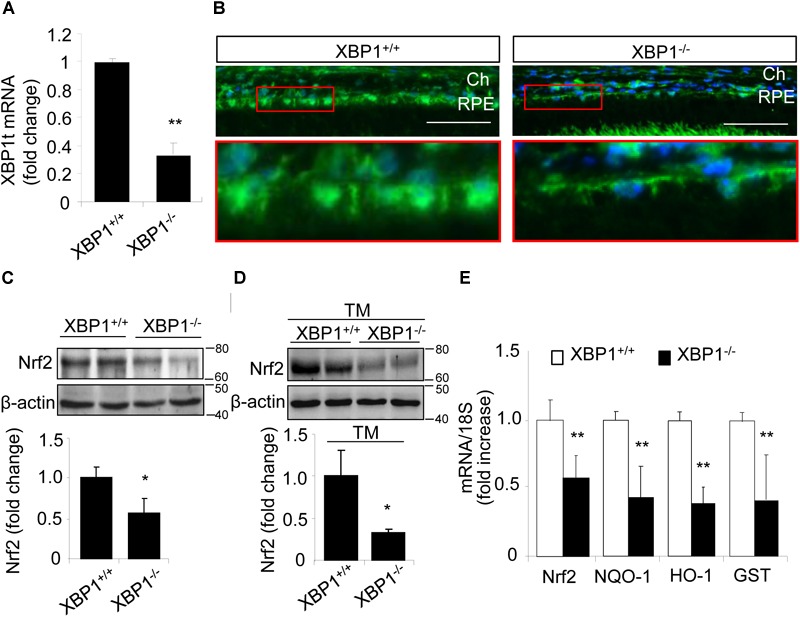
Decreased Nrf2 expression in the RPE of XBP1 KO mice. **(A)** Total XBP1 mRNA level in the WT and KO mice eyecups (including choroid and the RPE) was detected by real-time PCR (mean ± SD, *n* = 7 for WT, *n* = 5 for KO, ^∗∗^*p* < 0.01). **(B)** Retina sections were stained with anti-Nrf2 antibody (green) and DAPI (blue) was used to stain all nuclei. (Scale bar: 50 μm; Ch, choroid; RPE, retinal pigment epithelium). **(C)** Expression of Nrf2 in the WT and KO eyecups was determined by Western blot analysis and semi-quantified by densitometry (mean ± SD, *n* = 4, ^∗^*p* < 0.05). **(D)** Freshly isolated eyecups were incubated with 10 μg/ml tunicamycin for 6 h, and the protein level of Nrf2 was detected by Western blot and semi-quantified by densitometry (mean ± SD, *n* = 3, ^∗^*p* < 0.05). **(E)** mRNA levels of Nrf2, NQO-1, HO-1, and GST in the WT and KO eyecups was detected by real-time RT-PCR (mean ± SD, *n* = 5, ^∗∗^*p* < 0.01).

To determine if XBP1 regulates Nrf2 expression in the RPE, we examine the mRNA and protein levels of Nrf2 in the RPE/choroid complex from RPE-specific *XBP1* cKO mice. Successful downregulation of XBP1 in the RPE was confirmed by the significant reduction of total XBP1 mRNA level in the RPE/choroid complex of the cKO mice (Figure 1A). Immunohistochemical analysis showed that Nrf2 was expressed in the RPE of control mice, which was reduced in the cKO mice (Figure 1B). To quantify the change in Nrf2, RPE/choroid complex from the cKO mice or controls were subjected to Western blot analysis, which revealed a nearly 50% decrease in Nrf2 in the cKO (Figure 1C). We next examined whether the induction of Nrf2 by ER stress is affected in *XBP1*-deficient RPE. Eyecups (containing the RPE and choroid) from the cKO or control mice were exposed to 10 μg/ml of tunicamycin, a potent ER stress inducer, for 6 h. Induction of Nrf2 was evaluated by Western blot analysis. We found that Nrf2 expression was drastically decreased by 70% in *XBP1* cKO RPE/choroid complex compared to the controls after tunicamycin treatment (Figure 1D). The Nrf2 mRNA expression was also reduced by nearly 50% in *XBP1* cKO RPE/choroid complex (Figure 1E). To explore the consequence of *XBP1* knockout on downstream target genes of Nrf2, we measured the mRNA levels of NQO-1, HO-1, and GST. As expected, all three genes were suppressed after *XBP1* knockout (Figure 1E). These results suggest that XBP1 is required for both basal and ER stress-induced Nrf2 expression in the RPE.

### Nrf2 Expression Was Decreased in Primary RPE Cells Isolated From *XBP1* KO Mice

**FIGURE 2:**
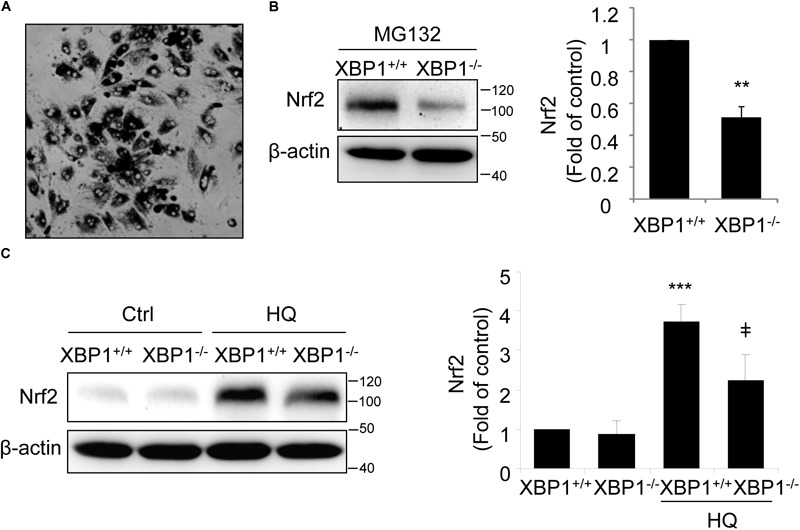
Impaired Nrf2 activation in primary RPE cells isolated from XBP1 KO mice. Primary RPE cells were isolated from WT and KO mice and cultured in a 12-well plate until confluence. **(A)** Phase contrast image of isolated RPE cells. **(B)** After proteasome inhibitor MG132 treatment (10 μM, 30 min), Nrf2 expression in primary RPE cells was detected by Western blot and semi-quantified by densitometry (*n* = 3 independent isolations, ^∗∗^*p* < 0.01). **(C)** Primary RPE cells were treated with a potent pro-oxidant, hydroquinone (HQ) (100 μM, 6 h), and Nrf2 expression was detected by Western blot (*n* = 3 independent isolations; ^∗∗∗^*p* < 0.001 vs. XBP1^+/+^ untreated control, ^‡^*p* < 0.05 vs. XBP1 ^+/+^ cells treated with HQ).

To further confirm the changes in Nrf2 expression in *XBP1*-deficient RPE, we isolated primary RPE cells from *XBP1* KO mice and WT mice. A representative image of mouse RPE cells is shown in Figure 2A. Given that the basal level of Nrf2 is low in normal cells due to a constant proteasomal degradation mediated by Keap1, we pretreated RPE cells with the proteasome inhibitor MG132 prior to harvesting cells for Western blot analysis. We found that in cKO RPE cells, Nrf2 protein level was about 50% of that in the WT cells (Figure 2B). We next examined whether induction of Nrf2 by stress is affected by exposing RPE cells to hydroquinone, a potent pro-oxidant identified in cigarette smoking that also induces ER stress (Chen et al., 2014). We found that hydroquinone-induced Nrf2 expression was significantly reduced in XBP1-null RPE cells (Figure 2C). These results further confirmed a role of XBP1 in Nrf2 regulation in the RPE.

### Overexpression of Spliced *XBP1* Increases Nrf2 Protein in ARPE-19 Cells

**FIGURE 3:**
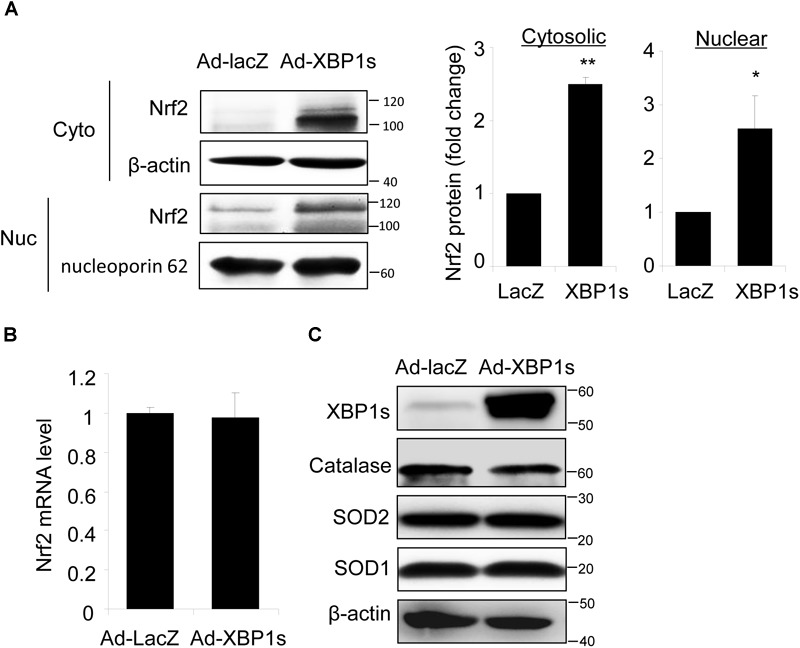
Overexpression of Spliced *XBP1* increased Nrf2 protein level in ARPE-19 cells. ARPE-19 cells were transfected with adenovirus overexpressing spliced *XBP1*. **(A)** Nrf2 expression in the cytosol and nucleus was measured separately by Western blot and semi-quantified by densitometry (mean ± SD, *n* = 3, ^∗^*p* < 0.05, ^∗∗^*p* < 0.01). **(B)** Nrf2 mRNA level was detected by real-time PCR (mean ± SD, *n* = 3). **(C)** Protein levels of XBP1, Catalase, SOD2, and SOD1 in whole cell lysate were detected by Western blot analysis.

Next, we determined whether overexpression of *XBP1* is sufficient to increase Nrf2 expression using a human RPE cell line, ARPE-19 cells transduced with adenovirus overexpressing spliced *XBP1* or *LacZ* as a transfection control. We found that Nrf2 protein levels in both cytoplasmic and nuclear fractions were significantly higher in Ad-*XBP1s* transduced cells compared to Ad-*LacZ* controls (Figure 3A). The transcription of Nrf2 was, however, not changed by overexpression of *XBP1s* (Figure 3B). Furthermore, we found that there is no change in the expression of other anti-oxidant genes including catalase, SOD2, and SOD1 (Figure 3C), indicating that overexpression of *XBP1s* did not simply increase oxidative stress. These results imply a potential role of spliced XBP1 in Nrf2 regulation.

### Inhibition of *XBP1* Splicing Reduces Nrf2 Expression in ARPE-19 Cells

**FIGURE 4:**
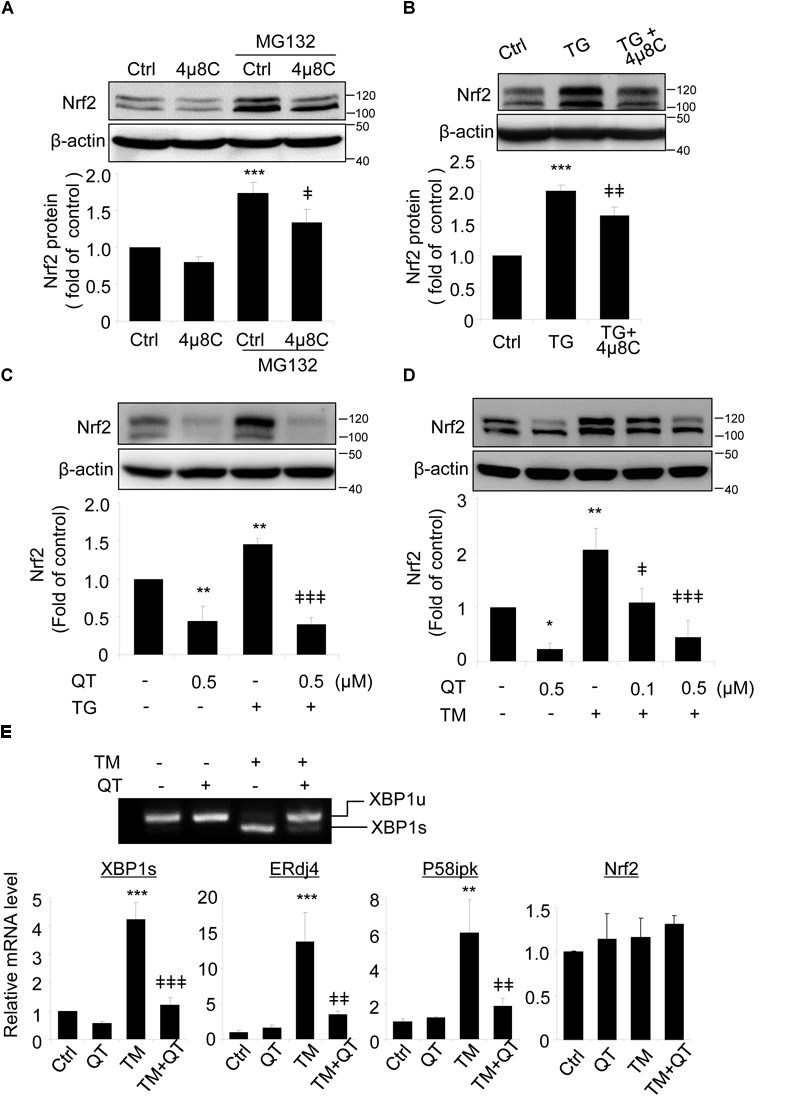
*XBP1* splicing inhibitors reduce Nrf2 expression in ARPE-19 cells. **(A)** ARPE-19 cells were treated with proteasome inhibitor MG132 (10 μM, 20 min) with or without pre-treatment of 4μ8C, an *XBP1* splicing inhibitor (25 μM, 8 h). **(B)** ARPE-19 cells were treated with ER stress inducer thapsigargin (TG) (10 μM) with or without 4μ8C (25 μM) for 8 h. Nrf2 expression was detected by Western blot and semi-quantified by densitometry (mean ± SD, *n* = 3, ^∗∗∗^*p* < 0.001 vs. control, ^‡^*p* < 0.05, ^‡‡^*p* < 0.01 vs. MG132 or TG treated cells). **(C,D)** ARPE-19 cells were treated with ER stress inducer thapsigargin (TG) (10 μM) or tunicamycin (TM) (50 ng/ml) with or without quinotrierixin (QT) for 8 h, Nrf2 expression was detected by Western blot and semi-quantified by densitometry. **(E)** ARPE-19 cells were treated with TM (50 ng/ml) with or without QT (0.5 μM) for 8 h, *XBP1* splicing was detected by RT-PCR. The levels of spliced XBP1, ERdj4, P58ipk and Nrf2 mRNA were determined by real-time PCR. (mean ± SD, *n* = 3, ^∗^*p* < 0.05, ^∗∗^*p* < 0.01, ^∗∗∗^*p* < 0.001 vs. control, ^‡^*p* < 0.05, ^‡‡^*p* < 0.01, ^‡‡‡^*p* < 0.001 vs. TM or TG treated cells).

Spliced XBP1 is the active form of XBP1, generated by splicing off a 26 bp fragment from XBP1 mRNA by inositol-requiring enzyme 1 (IRE1) during ER stress (Glimcher, 2010). To verify a role of spliced XBP1 in Nrf2 regulation, we pretreated ARPE-19 cells with 4μ8C, a specific inhibitor that binds to the RNase domain of IRE1α to suppress *XBP1* splicing (Cross et al., 2012; Ma et al., 2016), prior to incubation with ER stress inducer thapsigargin (TG). Upon 4μ8C treatment, Nrf2 protein level was reduced in ARPE-19 cells but this reduction only reached significance in the presence of MG132 (Figure 4A). Furthermore, induction of ER stress by TG increased Nrf2 levels and this increase was partially suppressed in cells pretreated with 4μ8C (Figure 4B).

To exclude potential off-target effects of 4μ8C, we pretreated ARPE-19 cells with quinotrierixin (QT), which specifically and selectively inhibits ER stress-induced XBP1 mRNA splicing (Kawamura et al., 2008a). Like 4μ8C, QT significantly and dose-dependently reduced the increase of Nrf2 levels induced by the ER stress inducers TG and TM (Figures 4C,D, respectively). Using RT-PCR and qPCR, we confirmed the significant downregulation of spliced XBP1 gene and two of its target genes, ERdj4 and P58ipk in QT treated cells (Figure 4E). Interestingly, neither TM nor QT altered the mRNA levels of Nrf2 (Figure 4E), suggesting that the regulation of Nrf2 by TM and spliced XBP1 may occur at the translational or post-translational levels.

**FIGURE 5:**
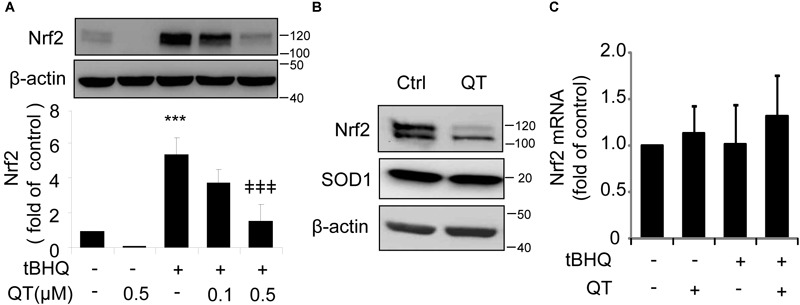
Quinotrierixin abolishes tert-Butylhydroquinone induced Nrf2 upregulation. **(A–C)** ARPE-19 cells were treated with tert-Butylhydroquinone (tBHQ) (10 μM) in the presence or absence of QT for 8 h. Nrf2 and SOD1 protein levels were detected by Western blot analysis **(A,B)** (mean ± SD, *n* = 3, ^∗∗∗^*p* < 0.001 vs. control, ^‡‡‡^*p* < 0.001 vs. tBHQ treated cells); Nrf2 mRNA level was detected by real-time PCR **(C)**.

To test this hypothesis, we treated ARPE-19 cells with tBHQ, which has been shown to induce Nrf2 activity by stabilizing ubiquitinated Nrf2 protein (Li et al., 2005), in the presence or absence of QT. We found that QT dose-dependently decreased tBHQ-induced Nrf2 stabilization (Figure 5A), but had no effect on SOD1 expression levels (Figure 5B). Furthermore, neither tBHQ nor QT altered Nrf2 mRNA levels (Figure 5C). These results support a post-transcriptional regulation of Nrf2 by XBP1.

### Quinotrierixin Suppresses the Basal Expression Levels of Nrf2 but Not the Half-Life

**FIGURE 6:**
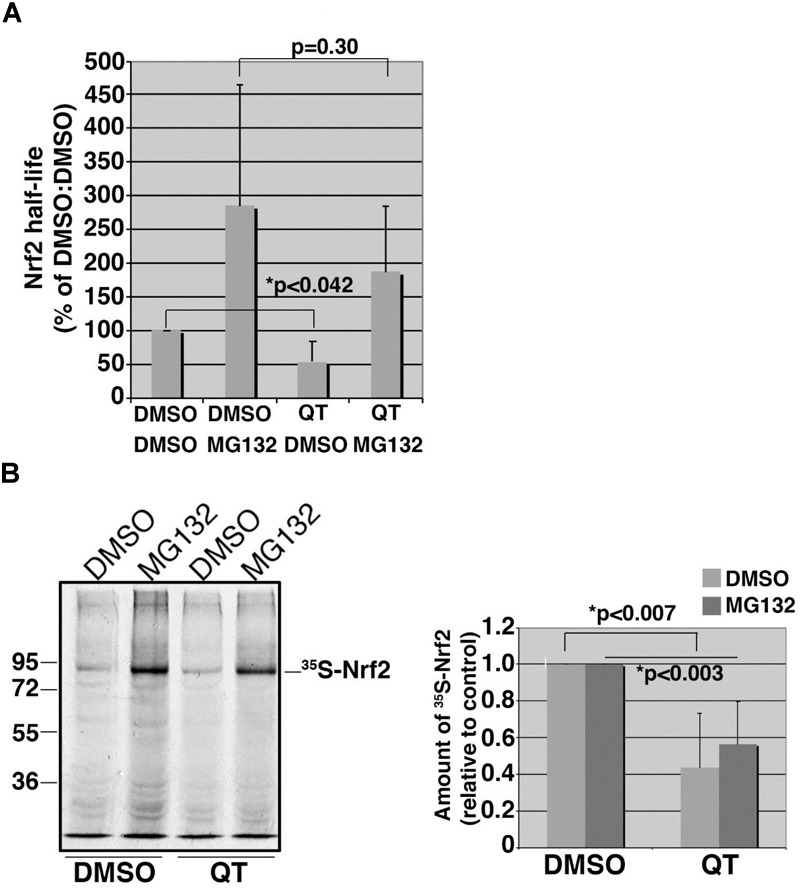
Quinotrierixin suppresses the basal expression levels of Nrf2 but not the half-life. **(A)** Graph of pooled data from three independent 35S-translabel, pulse-chase half-life experiments for endogenous Nrf2, comparing DMSO-treated cells to those treated with QT. Additional treatment with vehicle (DMSO) or MG132 was done to compare stabilized Nrf2. Half-life measurements were calculated using the formula, T1/2 = (t^∗^ln2)/(ln R0–ln Rt), where t = chase time, R0 = band intensity at t0, and Rt = band intensity at t. Two-tailed Student’s *t*-test indicates statistically significant difference in the starting amounts of Nrf2 (DMSO/DMSO vs. QT/DMSO) but no difference in half-life (DMSO/MG132 vs. QT/MG132). Asterisks denote statistical significance. **(B)** Representative fluorography showing the amount of endogenous, 35S-labeled Nrf2 stabilized and recovered by immunoprecipitation following an acute treatment with MG132 in cells pretreated with DMSO or QT. The migration of molecular weight markers is shown to the left of the fluorograph. Graph on the right shows data pooled from three independent experiments. Statistical significance was calculated by two-tailed Student’s *t*-test using the averages of three independent measurements for each treatment condition. Asterisks denote statistical significance.

To explore whether QT suppresses Nrf2 expression by accelerating protein degradation, we performed 35S-translabel, pulse-chase experiments in ARPE19 cells to measure the effect of QT on Nrf2 half-life. These studies revealed that the basal levels of Nrf2 were reduced by QT (Figure 6A; compare DMSO/DMSO vs. QT/DMSO) whereas the half-life was unchanged (Figure 6A; compare DMSO/MG132 vs. QT/MG132). Consistent with these half-life data, we found that the amount of newly synthesized Nrf2 protein (i.e., the amount synthesized in 40 min) was significantly reduced in QT-treated ARPE-19 cells versus control (Figure 6B). This reduction in Nrf2 synthesis was observed in both vehicle-treated cells as well as in the presence of MG132, to block proteasome-mediated degradation of the transcription factor (Figure 6B). These results indicate that QT reduces the *de novo* synthesis of Nrf2.

### Overexpression of *Nrf2* Had No Effect on XBP1 Expression in ARPE-19 Cells

**FIGURE 7:**
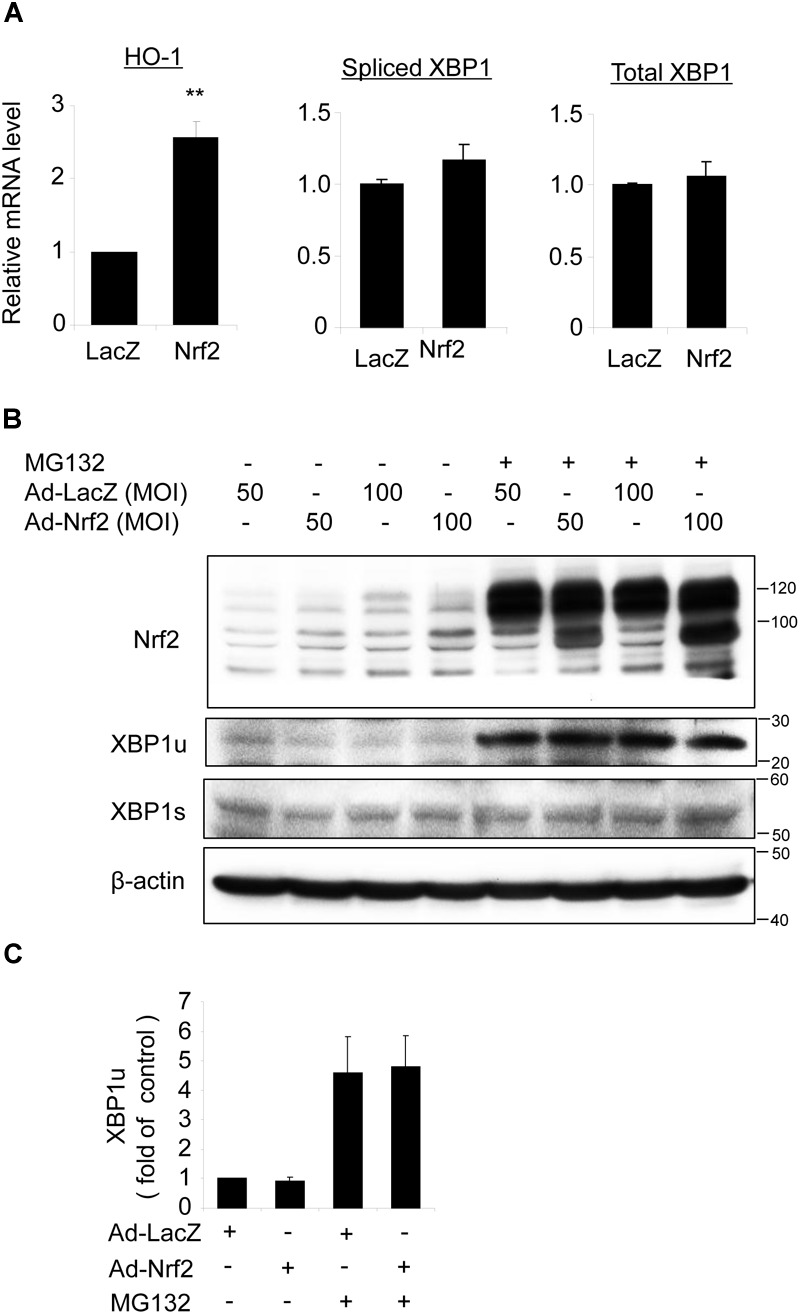
Overexpression of *Nrf2* does not alter XBP1 expression in ARPE-19 cells. **(A)** ARPE-19 cells were transfected with adenovirus overexpressing *Nrf2* or *LacZ* as control for 24 h, mRNA level of HO-1, spliced XBP1 and total XBP1 was determined by real-time RT PCR (mean ± SD, *n* = 3, ^∗∗^*p* < 0.01 vs. ad-LacZ transfected cells). **(B)** ARPE-19 cells were transfected with adenovirus overexpressing *Nrf2* or *LacZ* for 48 h, Nrf2, unspliced XBP1 (XBP1u) and spliced XBP1 (XBP1s) level was detected by Western blot analysis (representative of 3 independent experiments). **(C)** XBP1u expression was semi-quantified by densitometry (mean ± SD, *n* = 3).

To determine whether Nrf2 reciprocally regulates XBP1 expression, we transduced ARPE-19 cells with an adenovirus to overexpress *Nrf2*. Successful transduction was evidenced by increased expression of the Nrf2 target gene *HO-1* (Figure 7A) and enhanced Nrf2 protein level (Figure 7B). However, we found that neither total XBP1 nor spliced XBP1 mRNA levels were upregulated in Nrf2 overexpressed cells (Figure 7A). The protein levels of unspliced and spliced XBP1 were also unchanged with or without MG132 treatment (Figures 7B,C). These data suggest that Nrf2 does not induce XBP1 expression in RPE cells.

### Overexpression of *Nrf2* Protects Against Hydroquinone Induced DNA Damage in RPE Cells, but Is Insufficient to Reduce Cell Injury Caused by XBP1-Deficiency

**FIGURE 8:**
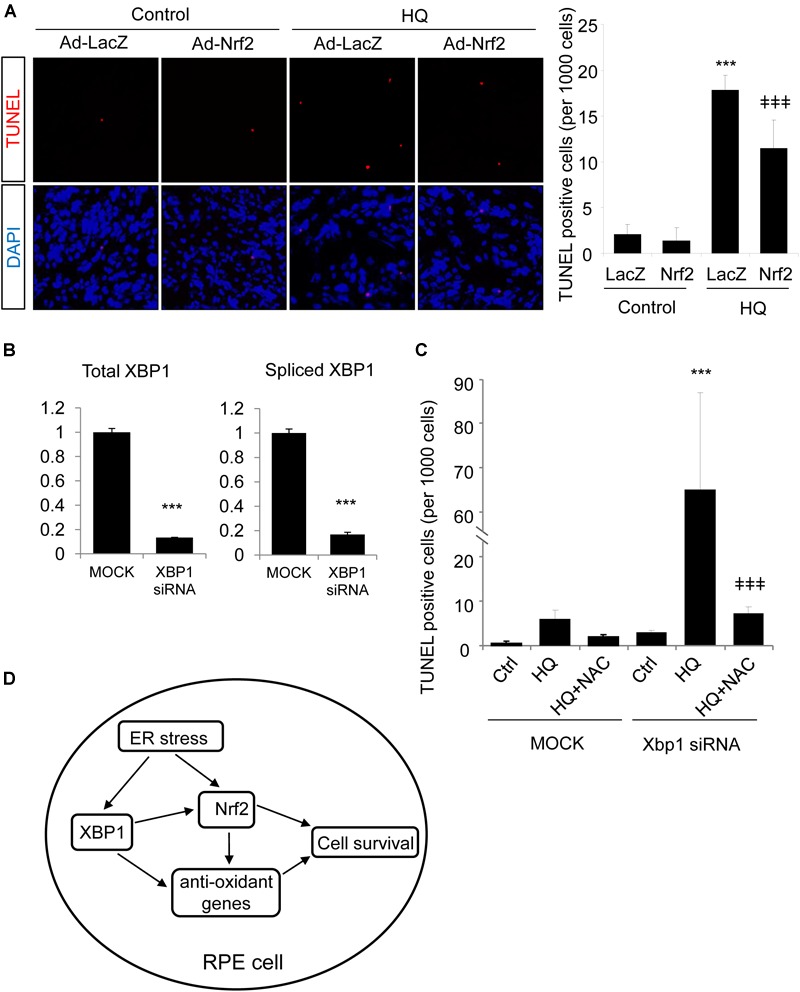
Overexpression of *Nrf2* protects ARPE-19 cells from hydroquinone induced injury. **(A)** ARPE-19 cells were transfected with adenovirus overexpressing *Nrf2* or *LacZ* as control for 24 h, then treated with a potent pro-oxidant, hydroquinone (100 μM, 24 h), for another 24 h. Cell DNA fragmentation was detected by TUNEL assay and quantified by cell counting (mean ± SD, *n* = 5 random 10× microscope field, ^∗∗∗^*p* < 0.001 vs. control, ^‡‡‡^*p* < 0.001 vs. Ad-*LacZ* transfected cells with HQ treatment). **(B)** ARPE-19 cells were transfected with *XBP1* siRNA or lipofectamine only (Mock) as control for 24 h, XBP1 knockdown efficiency was detected by real-time RT PCR (^∗∗∗^*p* < 0.001). **(C)** ARPE-19 cells were transfected with *XBP1* siRNA or lipofectamine only (Mock) as control for 24 h, then treated with hydroquinone (100 μM, 24 h) with or without N-Acetylcysteine (NAC) pre-treatment (1 mM, 2 h). Cell DNA fragmentation was detected by TUNEL assay and quantified by cell counting (mean ± SD, *n* = 5 random 10× microscope field, ^∗∗∗^*p* < 0.001 vs. untreated *XBP1* siRNA transfected cells, ^‡‡‡^*p* < 0.001 vs. *XBP1* siRNA transfected and HQ treated cells). **(D)** Schema of the regulation of Nrf2 by XBP1 in RPE cells. In the RPE, XBP1 upregulates the expression of Nrf2, promotes cell survival against oxidative stress. Loss of XBP1 leads to compromised Nrf2 synthesis, which promotes cell injury induced by oxidants. Meanwhile, XBP1 also regulates other anti-oxidative genes including Catalase, SOD1 and SOD2, which also contributes to anti-oxidative response of the RPE. Overexpression of *Nrf2* protects RPE cells from oxidants-induced injury, but could not compensate the loss of XBP1 on cell survival during oxidative stress.

Finally, we tested whether Nrf2 is sufficient to protect RPE cells from oxidative injury and to improve cell survival in *XBP1*-deficient cells. TUNEL staining was employed in this experiment. TUNEL assay detects not only fragmentation of genomic DNA associated with apoptosis, but also DNA damage associated with non-apoptotic events such as necrotic cell death (Loo, 2002). ARPE-19 cells were transduced with Ad-*Nrf2* or Ad-*LacZ*, and then exposed to HQ. In line with previous findings (Chen et al., 2014), we demonstrated that HQ induced a significant increase in cell DNA fragmentation (Figure 8A). Overexpression of *Nrf2* alleviated HQ-induced cell injury (Figure 8A). In order to determine whether overexpression of *Nrf2* protects XBP1-deficient cells from oxidant-induced injury, we co-transfected ARPE-19 cells with *XBP1* siRNA and Ad-*Nrf2*. Knockdown efficiency of *XBP1* was shown in Figure 8B. Expression of Nrf2 was detected by Western Blot (Supplementary Figure 1C). Phase contrast pictures demonstrated that XBP1 deficiency reduced cell viability and increased DNA damage indicated by TUNEL-positive cells (Supplementary Figure 1A). Interestingly, overexpression of *Nrf2* did not provide a protective effect in *XBP1* siRNA transfected cells (Supplementary Figure 1B). In contrast, pre-treatment of N-Acetylcysteine (NAC), a potent ROS scavenger, effectively reduced HQ-induced DNA fragmentation in *XBP1*-deficient cells (Figure 8C). These data indicated that overexpression of Nrf2 is insufficient to compensate the loss of XBP1 in RPE protection against strong pro-oxidants like HQ. The schema of the regulation of Nrf2 by XBP1 in RPE cells was shown in Figure 8D.

## Discussion

Increasing evidence suggest that the ER play an important role in regulation of cellular response to oxidative stress-induced damage in a variety of tissues and cell types (Cao and Kaufman, 2014). Previous studies from our group and several other laboratories demonstrate that oxidative stress and other genetic and environmental risk factors in AMD pathogenesis increase ER stress and activate the UPR in RPE cells (Cano et al., 2014; Chen et al., 2014; Kunchithapautham et al., 2014; Huang et al., 2015a,b). In cultured RPE cells, inhibition of ER stress or manipulating the UPR decreases ROS generation, reduces apoptosis, and improves cell survival, suggesting that ER stress activates an integrated signaling network governing redox homeostasis and cell survival (Huang et al., 2015b). Nrf2 is a central regulator of cytoprotective genes ubiquitously expressed in a variety of cell types (Tonelli et al., 2017). Expression of Nrf2 was found reduced in degenerating RPE cells in AMD, which leads to intensified oxidative stress and complement activation resulting in RPE injury, while overexpression of *Nrf2* protects against oxidative RPE damage (Cano et al., 2014; Huang et al., 2015b). Thus, understanding the regulation of Nrf2 is critical for developing new treatment to protect the RPE in disease conditions. In the present study, we demonstrate that XBP1, a major effector of the UPR, is required for ER stress- and oxidative stress-induced Nrf2 upregulation in RPE cells. This finding sheds new light on Nrf2 regulation in the RPE.

In recent years, the transcriptional factor XBP1 has been studied extensively. Various studies have demonstrated that XBP1 has an anti-apoptotic function (Romero-Ramirez et al., 2004; Kaser et al., 2008; Casas-Tinto et al., 2011). In MCF7 cells, XBP1 up-regulates the anti-apoptotic gene Bcl-2 and prevents antiestrogen therapy-induced cell death through inhibition of the mitochondrial apoptotic pathway (Gomez et al., 2007). In a previous study, we have shown that ablation of XBP1 in the RPE results in decreased expression of Catalase, SOD1, and SOD2, and increased ROS production in RPE-specific *XBP1* KO mice (Zhong et al., 2012). Using this mouse line and primary RPE cells derived from *XBP1* KO mice, we confirmed that loss of XBP1 downregulated expression of Nrf2 and its downstream genes including NQO-1, HO-1, and GST in the RPE. Furthermore, *XBP1*-deficient RPE cells show an impaired response to ER stress and oxidative stress inducers in stimulating Nrf2 protein production, suggesting that XBP1 could function as a critical regulator of cellular stress response in harnessing cytoprotective genes including Nrf2 to maintain the RPE function and survival under stress conditions.

In line with the central role of Nrf2 in anti-oxidant defense, expression of the transcription factor is regulated by sophisticated mechanisms at both transcriptional and post-transcriptional levels (Tonelli et al., 2017). In resting cells, Nrf2 is sequestered by Keap1 forming an inactive complex or targeted for proteasomal degradation (Surh et al., 2008). When challenged by oxidative stress, Nrf2 is stabilized and rapidly translocates into the nucleus to elicit a coordinated antioxidant response (Surh et al., 2008; Li and Kong, 2009). In addition to interacting with Keap1, Nrf2 is a substrate for the ER stress sensor, PKR-like ER kinase (PERK) (Schroder, 2008). Phosphorylation of Nrf2 by PERK disrupts its association with Keap1 resulting in Nrf2 nuclear accumulation and upregulation of antioxidant response genes (Verfaillie et al., 2010). Moreover, CHOP, the pro-apoptotic gene downstream of the PERK/eIF2α UPR pathway, has been reported by us to be essential for Nrf2 upregulation in cigarette smoking extract stimulated RPE cells (Huang et al., 2015b). Regulation of Nrf2 by UPR related genes demonstrate a crosstalk between ER stress and oxidative stress. In the present study, we found that the IRE/XBP1 branch of UPR also regulates Nrf2 expression. The data from our current study suggest that XBP1 activation may increase Nrf2 production via a post-transcriptional mechanism, because neither induction of ER stress nor an oxidative stress that activates XBP1, nor overexpression of spliced *XBP1* (the active form of XBP1), increase Nrf2 mRNA. Interestingly, in contrast to the drastically reduced Nrf2 expression in *XBP1*-null cells, overexpression of spliced *XBP1* only induced a modest but significant increase of Nrf2 protein in the nuclear and cytosolic fractions of RPE cells. Although this effect seems to be specific for Nrf2, i.e., without affecting other anti-oxidant proteins including SOD1, SOD2 and catalase, the discrepancy in the changes indicates the possible involvement of unspliced XBP1 in Nrf2 regulation. Unspliced XBP1 has been reported to be a negative feedback regulator of spliced XBP1 in Hela cells. Unspliced XBP1 forms a complex with spliced XBP1 and mediates rapid dissociation/degradation of the complex, thus shuting off the transcription of target genes during the recovery phase of ER stress (Yoshida et al., 2006). Moreover, unspliced XBP1 itself is a regulator of anti-oxidant genes. In HUVEC cells, overexpression of unspliced XBP1 promotes Nrf2 protein stabilization and nuclear translocation, and subsequent HO-1 induction (Martin et al., 2014). In mouse embryonic fibroblasts (MEFs), overexpression of unspliced XBP1 strongly increased catalase expression. The enhancing effect depends on CCAAT boxes and NF-Y-binding sites, while IRE activation is not necessary (Liu et al., 2009). Whether unspliced XBP1 is in part responsible for maintaining Nrf2 level in stressed RPE remains to be elucidated in future studies.

It is worth noting that although the predicted molecular weight of Nrf2 protein is 55–65 kDa according to its open reading frame size of ^∗^2.2-kb, the Nrf2 banding pattern in Western blots varies significantly as reported in the literature (Lau et al., 2013). This variation is thought largely attributable to the different degrees of ubiquitination of Nrf2 protein. In our Western Blots with mouse eyecups, tunicamycin-induced Nrf2 band was between 60 and 80 kDa, and this band was used for quantification. However, in cultured primary mouse RPE cells and ARPE-19 cells with chemical activation and adenoviral transduction, Nrf2 bands appeared to migrate to the position of 90–120 kDa. As discussed in detail by Lau et al. (2013), Nrf2 bands at 95–110 kDa should be considered as biologically relevant Nrf2. Therefore, we included the bands within this molecular weight range for Nrf2 quantification.

One of the XBP1 inhibitors used in this study, QT, is a member of the triene-ansamycin group antibiotics. It was first identified by the Tashiro group in 2007, as a specific inhibitor of ER stress-induced XBP1 mRNA splicing (Kawamura et al., 2008a). QT dose-dependently inhibits TG-induced *XBP1* splicing in Hela cells with an IC50 of 0.067 μM (Kawamura et al., 2008b). However, a later study from the same group demonstrated that QT inhibits the protein production of UPR genes, including the 78-kDa glucose-regulated protein (GRP78), C/EBP homologous protein (CHOP), ERdj4 and P58ipk (Yamamoto et al., 2011). In our current study, we found that QT significantly reduced Nrf2 protein levels in non-stimulated RPE cells and blunted the increase of Nrf2 protein induced by ER stress or oxidative stress. Interestingly, QT showed no effect on SOD1 expression in the RPE. Further mechanistic experiments revealed that QT treatment does not seem to alter the transcription or increase protein degradation of Nrf2. Thus, it indicates that likely QT reduces Nrf2 through inhibition of protein synthesis. This finding appears to be consistent with the observations by the Tashiro group. However, whether QT reduces Nrf2 protein translation and how QT selectively inhibits Nrf2 but not other antioxidative proteins are yet to be determined.

Another interesting finding of this study is that overexpression of Nrf2 was not able to rescue *XBP1*-deficient RPE cells challenged with HQ. One possible explanation is that deprivation of XBP1 negatively influences the expression/function of multiple anti-oxidant genes such as catalase, SOD1, and SOD2 and the consequent reduction in the anti-oxidant capacity leads to increased cell injury when challenged by HQ. Supplementing with additional Nrf2 was also not sufficient to reverse cellular redox disturbance, thus cell injury in *XBP1*-deficient cells was not attenuated. In contrast, pre-treatment of N-Acetylcysteine (NAC) effectively protected cells from HQ-induced injury, suggesting that scavenging ROS can compensate for the loss of XBP1 in the RPE cells, furthering supporting the role of XBP1 in regulating the anti-oxidative response in the RPE.

Taken together, our results demonstrate that in the RPE, XBP1 is required for Nrf2 expression. Deficiency of XBP1 results in a decreased anti-oxidant response that contributes to oxidative injury of the RPE, which is highly relevant to the pathogenesis of AMD.

## Data Availability Statement

The raw data supporting the conclusions of this manuscript will be made available by the authors, without undue reservation, to any qualified researcher.

## Author Contributions

SZ and JW conceived and supervised the experiments, interpreted the data, and wrote the manuscript. QY designed the experiments, participated in experiments, and interpreted the data. CC, YZ, KP, and SP performed the experiments, analyzed the data, and wrote the manuscript. All authors have read and approved the final version of the manuscript.

## Conflict of Interest Statement

The authors declare that the research was conducted in the absence of any commercial or financial relationships that could be construed as a potential conflict of interest.
