# Supplementary material for: Regulation of Nrf2 by X Box-Binding Protein 1 in Retinal Pigment Epithelium
Source: Front Genet. 2018 Dec 20;9:658. doi: 10.3389/fgene.2018.00658 (PMC6306429; doi:10.3389/fgene.2018.00658)
Supplement: Supplementary file 1 [file Image_1.pdf]

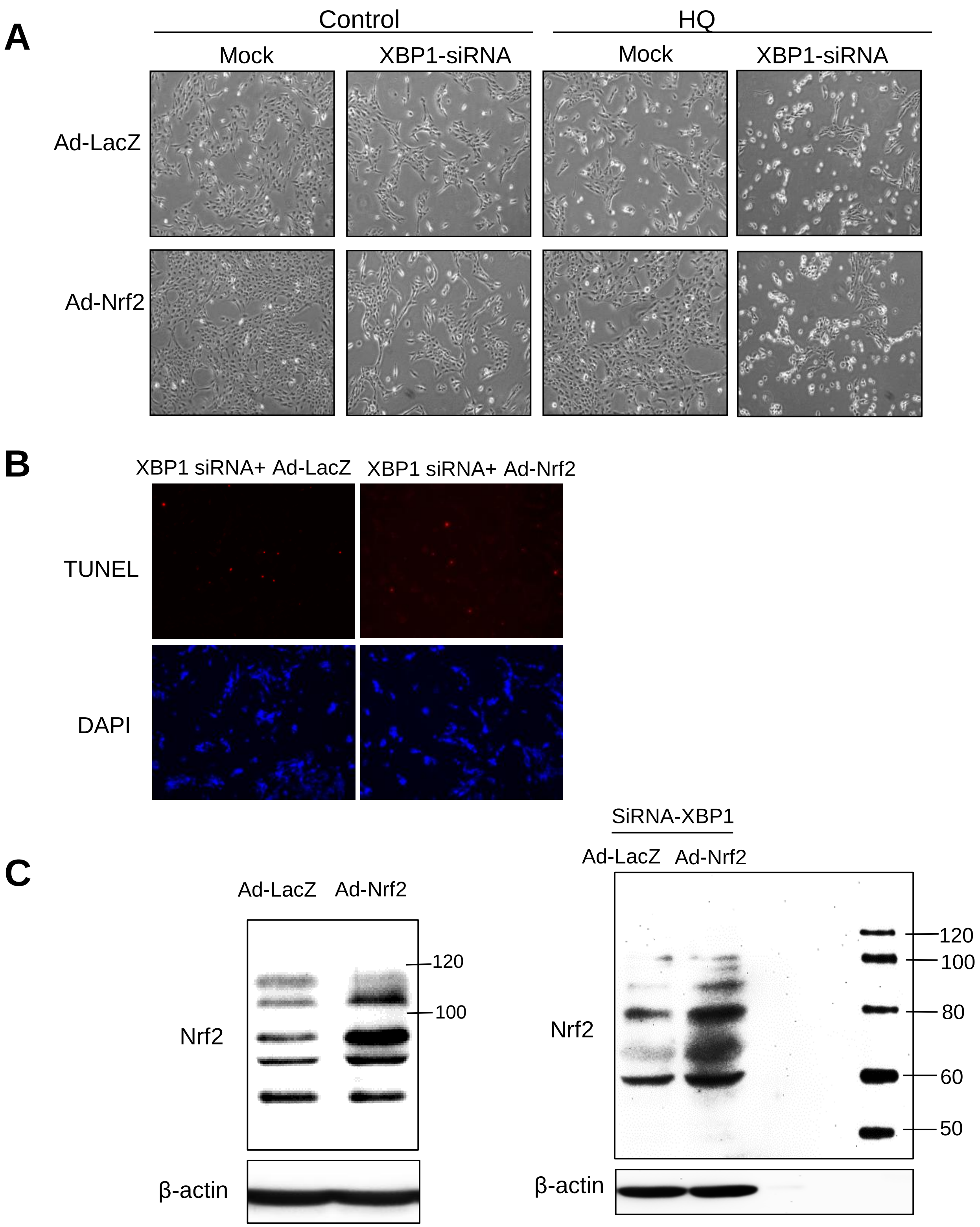

Supplementary Figure 1. Loss of XBP1 exacerbated hydroquinone induced cell death in ARPE-19 cells, and overexpression of *Nrf2* exhibited no rescue. ARPE-19 cells were transfected with *XBP1* siRNA or lipofectamine only (Mock) as control for 24 h, then infected with adenovirus overexpressing *Nrf2* or *LacZ* as control for another 24 h. Then cells were treated with hydroquinone (100 μM, 24 h), and cell death and DNA damage was detected by phase contrast microscopy **(A)** and TUNEL assay **(B)**, respectively. The level of Nrf2 expression was detected by Western Blot **(C)**.
